# Development of Rule-Based Diagnostic Automation Technology for Elevator Fault Diagnosis

**DOI:** 10.3390/s26010223

**Published:** 2025-12-29

**Authors:** Sangyoon Seo, Jeong jun Lee, Dong hee Park, Byeong keun Choi

**Affiliations:** 1 Research & Development, Korea Elevator Safety Agency, Geochang 50148, Republic of Korea; mulphy@koelsa.or.kr; 2 Department of Mechanical Engineering, Ulsan College, Ulsan 44610, Republic of Korea; jjlee4@uc.ac.kr; 3 DAVISS Inc., Jinju 52828, Republic of Korea; daviss.dhp@gmail.com; 4 Department of Energy and Mechanical Engineering, Gyeongsang National University, Tongyeong 53064, Republic of Korea

**Keywords:** elevator, fault diagnosis, rule-base system, automation, signal recognition

## Abstract

Elevators are critical vertical transportation systems in modern urban infrastructure; however, their intricate mechanical and electrical configurations render them highly susceptible to safety-critical failures. Although various automated diagnostic techniques have been proposed, many data-driven approaches exhibit limited generalizability due to their insufficient consideration of physical fault mechanisms and strong dependence on facility-specific training data. To overcome these limitations, this study presents a rule-based automated diagnostic framework for elevator state recognition that prioritizes reliability, real-time performance, and interpretability. The proposed approach explicitly integrates physically meaningful fault characteristics and dominant frequency components into the diagnostic process, and employs predefined expert rules derived from established standards to classify fault states in an automated manner. The effectiveness of the proposed method is verified using real operational data collected from an in-service elevator, demonstrating improved diagnostic accuracy and computational efficiency compared to conventional manual inspection procedures. The proposed framework provides a practical and scalable solution for intelligent elevator condition monitoring and is expected to serve as a foundational technology for future smart maintenance and preventive safety systems.

## 1. Introduction

In modern society, elevators have become an essential means of transportation for moving people and goods in various buildings, such as high-rise buildings and apartments. As urbanization and the construction of high-rise buildings continue, the number of installed elevators has been steadily increasing. Consequently, elevators are now regarded not merely as simple transportation devices but as critical infrastructure responsible for the efficient operation and convenience of modern buildings. Currently, elevator facility management is conducted through self-inspections in accordance with the Elevator Safety Management Act [1,2,3]. However, in practice, a large portion of elevator maintenance still relies on breakdown maintenance approaches, in which problems are addressed only after failures have occurred [2,3]. Moreover, most elevator self-inspections are performed based on inspectors’ experience and visual checks. As a result, quantitative analysis of sensor signals generated during elevator operation—such as vibration, noise, and electrical current—is rarely conducted, making it difficult to accurately evaluate early signs of faults. According to a survey by the National Elevator Information Center, the number of elevator fault-related complaints has steadily increased over the past five years. This trend indicates clear limitations in existing elevator management and inspection methods. Since elevator faults are directly related to user safety, unexpected failures not only cause inconvenience but may also pose serious safety risks to passengers in severe cases [3,4,5,6].

In response to the growing demand for improved fault management strategies, AI-based facility diagnostic systems utilizing Fourth Industrial Revolution technologies have already been introduced in various industrial fields. Such technologies can automatically analyze facility conditions and detect abnormalities, thereby significantly improving maintenance efficiency. In particular, they enable automated diagnosis even in situations where expert resources are limited, and they allow objective and data-driven assessment of equipment conditions. Although the application of such AI-based diagnostic systems is also highly desirable in the elevator industry, there are still substantial challenges in directly applying machine learning or deep learning techniques to elevator facilities [7,8,9,10,11,12,13,14,15].

First, the performance of machine learning–based diagnostic models strongly depends on the quantity and quality of available training data. However, it is extremely difficult to obtain sufficient fault or defect data from elevator systems, as most elevators operate under normal conditions for long periods and fault data can only be collected when actual breakdowns or accidents occur [15]. Artificially generating fault data or acquiring it through experimental setups is both time-consuming and economically burdensome, and such data often fail to accurately reflect real operating environments. Consequently, data scarcity and imbalance remain critical obstacles to the practical deployment of data-driven diagnostic models for elevators.

In addition to data-related limitations, existing AI-assisted elevator diagnostic studies and industrial applications—particularly those based on rule-based or vibration-based approaches—have often remained at a simple alarm level, relying on threshold-based judgments of single signal indicators or ride comfort criteria [16]. These limitations are closely related to the inherent structural characteristics of elevator systems, which differ fundamentally from those of conventional rotating machinery. Elevators consist of unique structural components such as guide rails, suspension ropes, and a moving car, and elevator-specific fault types—including guide rail misalignment and rope tension imbalance—frequently occur as a result. However, existing diagnostic approaches have not sufficiently incorporated these structural features and associated fault mechanisms, making it difficult to accurately distinguish fault types and ensure consistent diagnostic performance [17,18,19].

Furthermore, elevator facilities exhibit significant diversity depending on installation environments and operating conditions. Facility specifications vary according to building type (e.g., residential, office, or commercial buildings), number of floors, rated speed, load capacity, and drive mechanisms. This heterogeneity leads to differences in signal characteristics and data distributions across facilities, posing fundamental limitations for training generalized machine learning models. Developing facility-specific models requires separate data collection and model training processes for each elevator, resulting in increased time, cost, and operational complexity. At the same time, differences in data distributions make it difficult to construct a single generalized diagnostic model applicable to a wide range of elevator systems [18,20].

Recently, physics-guided and physics-informed learning approaches have been proposed as alternatives to mitigate data scarcity issues in data-driven models. Methods such as Physics-guided Degradation Trajectory Modeling and Physics-Informed Neural Networks (PINNs) aim to enhance generalization performance by embedding physical knowledge into the learning process. While these approaches are promising, their application to elevator systems remains challenging [18,19]. Elevators are characterized by complex system configurations, a large number of components, diverse installation conditions, and strict safety regulations. Accurately modeling such systems requires high computational complexity and detailed physical modeling, and most existing studies have been limited to simulation environments or controlled experimental settings. Therefore, despite their academic significance, physics-based approaches have yet to be validated as immediately applicable diagnostic automation solutions in real-world elevator operations [20].

Accordingly, this study focuses on developing a rule-based diagnostic automation technology for elevator fault diagnosis that addresses the limitations of both data-driven and physics-based approaches. Rule-based diagnostic systems perform diagnosis by responding to predefined attributes derived from expert knowledge, enabling expert-level diagnostic decisions without the need for large-scale training data [21,22]. Unlike conventional rule-based approaches that rely on simple threshold-based alarms, the proposed method systematically incorporates multidimensional diagnostic attributes, including signal magnitude, frequency components, repetitiveness, and abnormal event characteristics.

Specifically, this study aims to extract diagnostic variables from time-domain vibration waveforms and frequency-domain FFT spectra that are commonly used by elevator diagnostic experts. These variables are then formalized into diagnostic rules based on their physical meanings and signal characteristics. By responding to combinations of these attributes, the proposed system can distinguish between normal and faulty states and identify fault patterns across mechanical drive components and structural elements of the elevator system. Through this approach, the proposed rule-based diagnostic automation framework not only compensates for the data scarcity problem inherent in existing AI-based technologies but also reduces dependence on subjective expert judgment. Ultimately, the proposed method is expected to significantly enhance the reliability and efficiency of elevator maintenance by providing an interpretable, practical, and immediately applicable diagnostic solution for real-world elevator facilities.

## 2. Elevator Fault Characteristics Analysis

### 2.1. Structural Characteristics of Gearless Type Elevator

**Figure 1:**
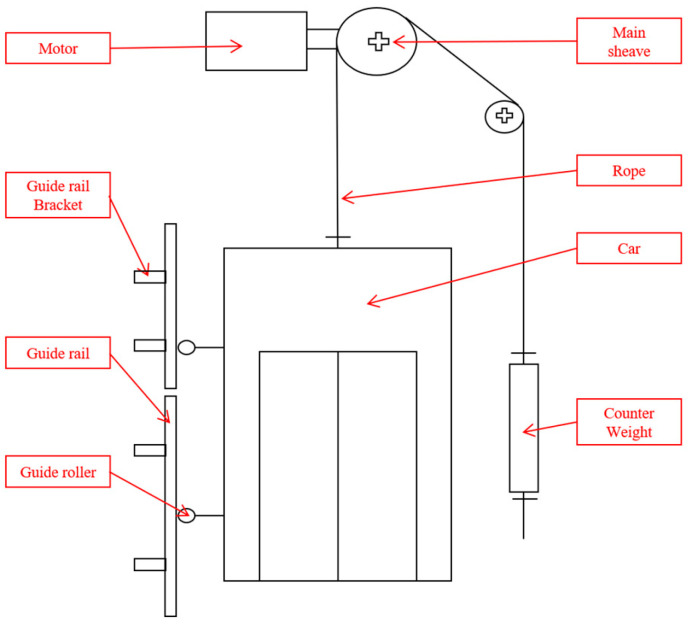
Schematic Diagram of Elevator.

An elevator transmits power by rotating a sheave with a motor and transports passengers or goods by moving the car up and down with a connected rope. Figure 1 is a schematic diagram of the elevator structure, showing the main components that affect operation and ride comfort. The main components include the motor, main sheave, and guide rail, and many other parts operate in a related manner. These parts each perform their own individual roles, but in the case of the driving part, the sheave is directly connected to the motor shaft and operates in interaction with each other. Accordingly, if a fault occurs in one part, it will affect other connected parts, not only causing discomfort in the ride, but may even lead to a stop in operation. For example, if a fault occurs in the motor, it will affect the main sheave and this effect will be transmitted, causing vibrations inside the car.

In addition, the guide rail plays a role in guiding the car so that it can move stably, but if it is misaligned or fails to perform its role as a support, a transient signal is generated inside the car. This means that the interconnectivity between each component requires a complex fault diagnosis rather than simply deriving a single fault. In this way, for maintenance and safety assessment, it is recommended to individually evaluate the condition of major components and comprehensively analyze the interactions between them. In other words, multiple faults can occur simultaneously, so it is important to understand the characteristics of each component and the mechanism for the fault, perform an analysis, and then present the overall fault to maintain reliability and safety [17,23,24,25,26].

### 2.2. Fault Diagnosis Using Vibration Signals

While international standards exist for the diagnosis of rotating machinery, diagnosis using the vibration signal of elevators is not actively being conducted due to the lack of clear criteria for vibration values. ISO 8100-34 [27] specifies that when evaluating vibration magnitude, the Peak-to-Peak value of the raw signal and the A95 Peak-to-Peak value excluding 5% of the maximum value should be checked. In addition, Korea Land & Housing Corporation sets and uses the Peak-to-Peak or A95 standard for lateral vibration, 15 gal for LPF (Low pass filter, 10 Hz) application, and 20 gal for longitudinal vibration (direction of operation) as the criteria for vibration values [25]. Considering these factors, this study aims to evaluate the condition of the elevator by utilizing the amplitude in each direction based on the Peak-to-Peak value rather than the A95 when diagnosing a fault. This is because the A95 and LPF (10 Hz) values have the characteristic of excluding transient signals or high-frequency signals occurring above 10 Hz, respectively, making it difficult to effectively diagnose faults containing sudden impulse components and high-frequency components [26,28].

**Figure 2:**
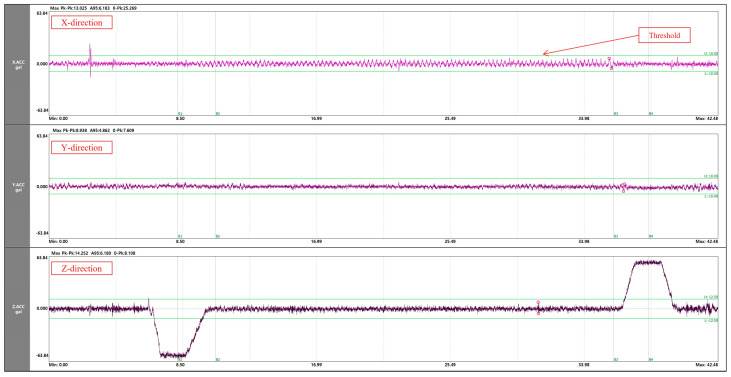
Measurement Sample of Elevator Vibration Signals.

When performing elevator fault analysis using vibration signals, all X, Y, and Z directions must be considered as mentioned above. In particular, since vibration signals have directionality, different characteristics appear depending on each direction [17,18,28,29]. Figure 2 shows the results of obtaining ride comfort data using a 3-axis acceleration sensor. It can be confirmed that rising and falling sections appear on the Z-axis, while this does not occur on the X and Y-axes, and different characteristics appear. It is necessary to perform a diagnosis considering these characteristics, and when the direction of occurrence of the fault and the parts are considered, the diagnosis region can be divided into the following five regions: the driving part that transports the elevator, the motor, the guide rail, the car, and the guide roller.

#### 2.2.1. Driving Part (Main Sheave)

**Figure 3:**
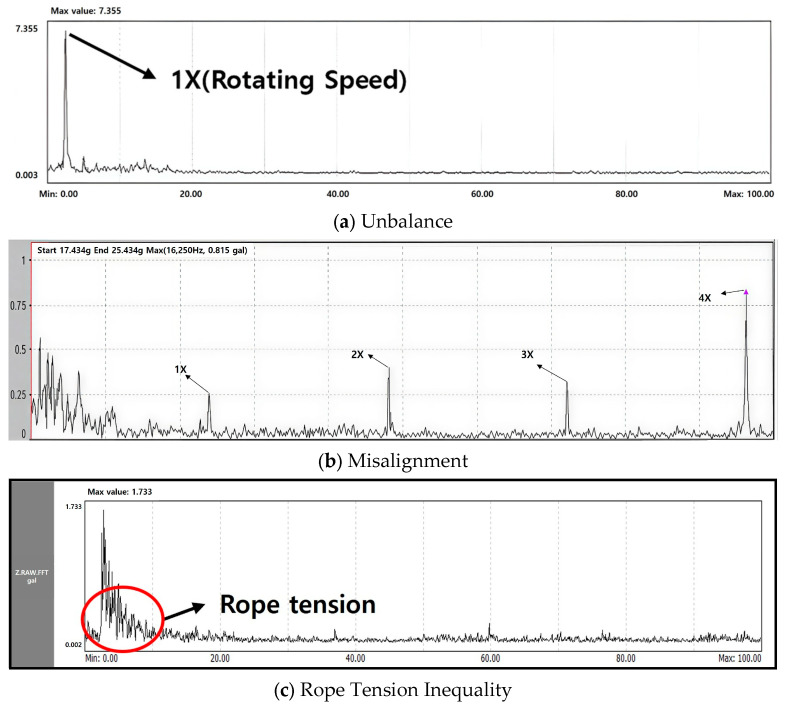
Example of Faults Related to Main Sheave.

The main role in moving the elevator up and down is played by the driving part. It is the main cause of lowering the operational stability of the elevator and can cause faults such as sheave unbalance and misalignment. Faults related to the driving part have predominant frequency components in the up and down directions. Figure 3 is a diagram showing faults that can occur in a sheave. When unbalance occurs, the 1X component appears high, and misalignment causes harmonic components such as 2X and 3X to appear. This is similar to the fault characteristics that occur in general rotating machinery. In the case of rope tension faults, it is confirmed that the rope tension changes continuously over a long period of time, resulting in a haystack energy in the low-frequency range.

#### 2.2.2. Motor

**Figure 4:**
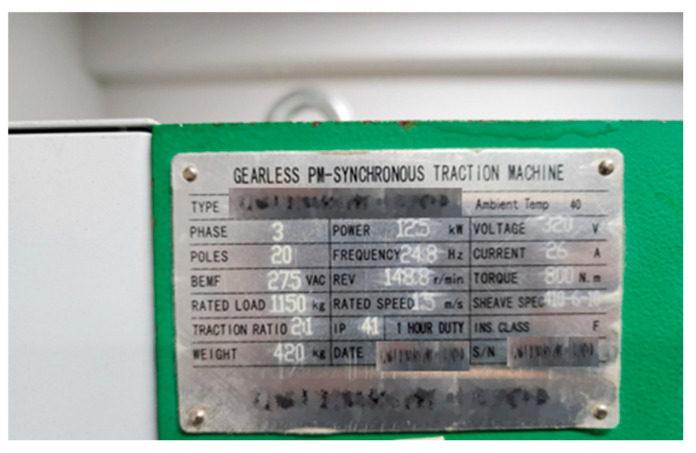
Motor Specification.

**Figure 5:**
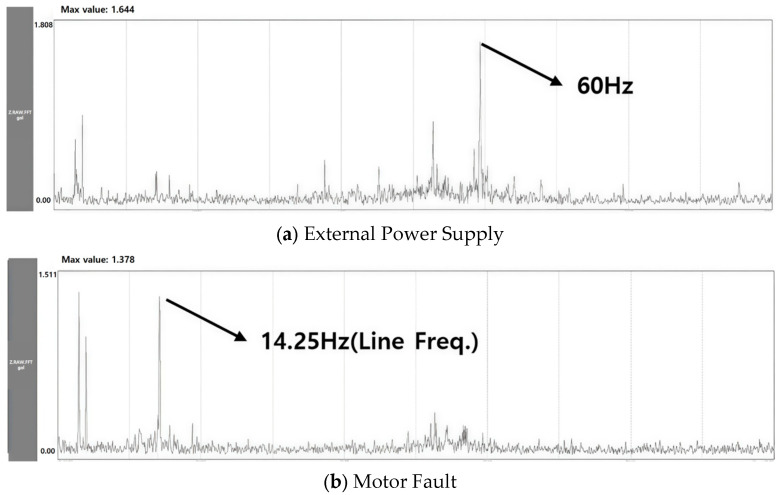
Comparison of External Power Supply & Motor Fault Signals.

The motor is the main drive unit of the elevator and is a key component responsible for acceleration and deceleration of the elevator. Accordingly, although it is part of the driving part, there are many faults in the motor that occur independently, such as voltage unbalance or coil and slot defects, so it is necessary to separate them and evaluate them additionally. Since the motor is connected to the sheave and receives a load in the Z direction, it exhibits characteristics related to the Z-axis, just like the driving part. An additional consideration is that the line frequency is generated after passing through the inverter from the power supply, so this must be distinguished when diagnosing a fault. Figure 4 shows information about the motor, which is summarized in Table 1. When the power supply is 60 Hz, the rated frequency is designed to rotate at 24.8 Hz. Figure 5 shows the faults caused by the synchronous motor and external power components. If the frequency component, such as the power supply, is a fault caused by an external source and not a fault in the motor, this must be taken into consideration.

#### 2.2.3. Guide Rail

**Figure 6:**
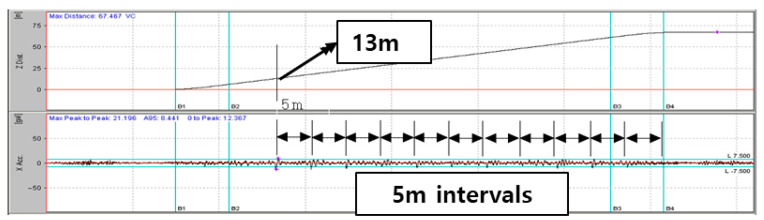
Example of Guide Rail Fault (Misalignment).

The guide rail performs the function of guiding the car and counterweight to move stably and ensures the straight movement of the elevator. The guide rail supports the elevator in the X and Y directions, and if there is a fault or a misalignment in the guide rail support, a peak component may occur due to the bracket location or the installed rail spacing. Figure 6 is an example of a guide rail misalignment fault. Since guide rails are connected at 5 m intervals in Korea, it is confirmed that impulse components appear at 5 m intervals. It is necessary to explore whether the impulse component appears at the same location based on the pit depth and the initial guide rail length.

#### 2.2.4. Car

**Figure 7:**
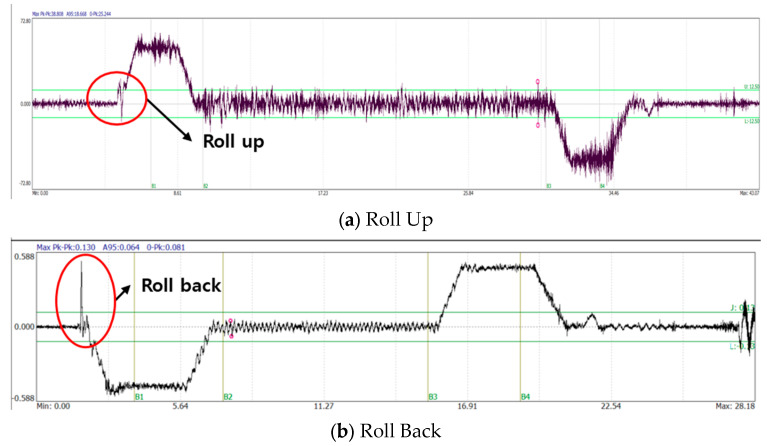
Example of Car Faults (Roll Up & Back).

A car is a space for transporting passengers or goods, and is the part where passengers directly experience the ride quality. If there is a malfunction in the rope or operation, it may feel like it is bouncing or it may start moving in the opposite direction and then starting again. The resulting vibrations can cause discomfort and anxiety to passengers, and generate transient signals in the Z direction, the direction of operation. Figure 7 shows the measurement results for Roll up and Roll back. The characteristic does not appear in frequency, and the impulse component occurs just before acceleration or deceleration.

#### 2.2.5. Guide Roller

**Figure 8:**
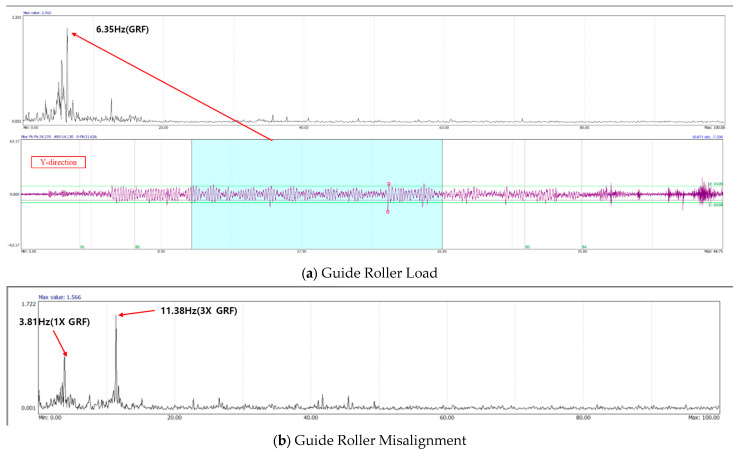
Example of Guide Roller.

Finally, the guide rollers serve to reduce friction between the guide rail and the car, thereby helping to ensure smooth movement. However, in the event of load faults or misalignment of the guide roller, a frequency component is generated as much as the rotation of the guide roller, and this is the same as the guide rail and occurs in the X and Y directions. Figure 8a is the FFT spectrum displayed when a load is applied to the guide rail, and the GRF (Guide Roller Frequency) 1X component is dominantly high. The calculation of the corresponding frequency component can be done using Equation (1) because it is the number of rotations of the guide roller. Additionally, if a multiple component of GRF is dominant as in Figure 8b, it indicates misalignment of the guide roller.

(1) $$GRF=v\_car/(\pi D_{gr})$$

## 3. Development of Rule-Based Diagnostic Automation Technology

### 3.1. Establishing the Target of Recognition

Rule-based diagnosis is a system that creates a decision tree based on expert knowledge and an inference engine, allowing non-experts to arrive at the same conclusions as experts through responses to attributes [30,31,32]. Current rule-based diagnosis requires users to respond to attributes based on information presented in the time waveform or FFT spectrum, but if information is automatically recognized and responses are provided, individual user errors or human errors can be reduced [21,22,23]. In other words, rather than technologies that require a large amount of data, such as machine learning and deep learning, it is more appropriate to combine fault patterns that may appear using the specifications of machinery with technologies using the long-term experience of experts to apply them to actual sites. Therefore, in this study, we aim to generate attributes using established elevator fault components and expert knowledge base and develop recognition technology and automatic response technology.

**Figure 9:**
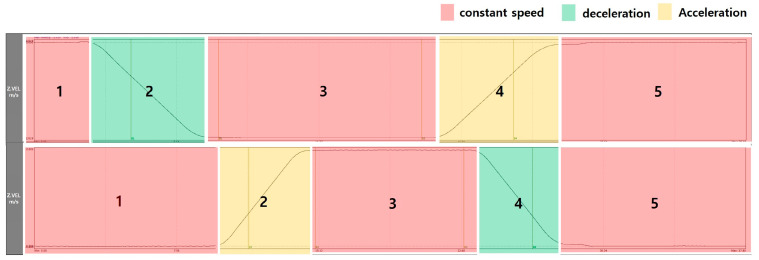
Example of Elevator Speed Zone.

Figure 9 is a diagram showing the speed of an elevator. The region is divided into a total of 5 regions: stop, acceleration or deceleration, constant speed, deceleration or acceleration, and stop state. As mentioned in Region 2, it is appropriate to diagnose the sheaves, motors, and guide rollers in Region 3, which is a constant speed section, because faults occur during operation. And faults such as Roll up & Roll back are best diagnosed in Regions 1 and 2, which transition from a stop to an acceleration or deceleration section. Accordingly, in this study, faults were recognized by dividing them by region.

To classify the condition of a fault, a pattern or characteristic representing each condition must be selected as a recognition target. Table 2 summarizes the characteristics of each fault, and the domain is divided into time waveform and FFT spectrum. In the time domain, it is divided into two characteristics depending on the fault. Roll up & Roll back are characteristics that drive in reverse from the start point of operation on the Z-axis and then drive in the forward direction again. Next, if there occurs a misalignment or a fault in the support part at the guide rail joint, there is a characteristic that an impulse component occurs at a certain interval inside the car on the X-axis or Y-axis. These two characteristics need to be recognized as impulse components occurring in the time waveform, and it must be considered that they occur in different regions. In the FFT spectrum, it is necessary to determine the occurrence of single frequency and harmonics components that appear according to operation and fault conditions, and of haystack energy caused by random excitation. In summary, it is necessary to recognize a total of four components as follows: the impulse component in Regions 1 & 2, the impulse component that appear at regular intervals, the single frequency component, and the haystack energy component.

### 3.2. Development of Recognition Technology

#### 3.2.1. Frequency Component Recognition

**Figure 10:**
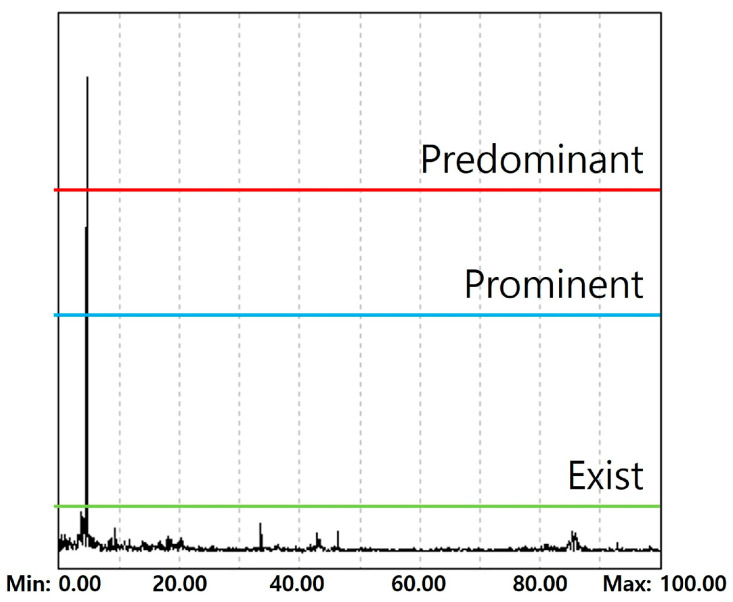
Discrimination Criteria.

In frequency-domain–based diagnosis, the occurrence of a fault cannot be determined by frequency information alone; both the frequency location and the magnitude of the spectral component must be considered. For this purpose, amplitude-based criteria are introduced to categorize the significance of frequency components in the FFT spectrum. As shown in Figure 10, the amplitudes of frequency components observed in the Z-axis spectrum are classified into three levels: Predominant (≥5 gal), Prominent (≥2.5 gal), and Exist (≥1 gal). The Predominant level of 5 gal was selected with reference to the vibration magnitude benchmarks discussed in Section 2.2. Considering that allowable ride-comfort levels are on the order of 15–20 gal peak-to-peak, a single spectral component reaching approximately 5 gal represents a substantial portion of the total admissible vibration. Such a component clearly stands out from background vibration and is highly likely to be associated with an abnormal mechanical condition rather than normal operation. Therefore, frequency components exceeding 5 gal are treated as dominant diagnostic features.

The Prominent (2.5 gal) and Exist (1 gal) thresholds were set proportionally lower, reflecting expert knowledge of elevator maintenance practice. Experienced diagnosticians typically evaluate fault conditions by sequentially examining dominant frequency peaks, followed by moderately strong and then minor components that may support or refine the diagnosis. While frequency components below the Prominent level often have limited standalone diagnostic impact, the Exist level is retained to ensure that weaker but characteristic frequencies are not overlooked when additional contextual evidence is required. It should also be emphasized that these amplitude levels do not represent safety limits or immediate failure criteria. Instead, they serve as internal diagnostic categories that trigger further analysis within the proposed system. As additional data from diverse elevator types and operating environments become available, these threshold values can be refined in future work.

**Figure 11:**
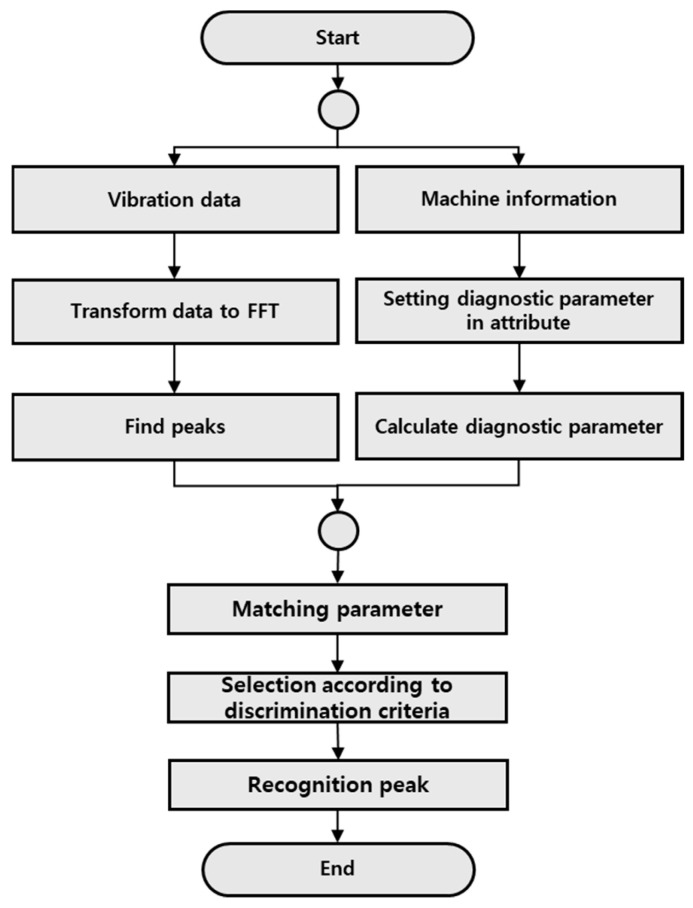
Flowchart of Frequency Component Recognition Algorithm.

Figure 11 is a diagram showing an algorithm for recognizing peaks. Based on facility and operation information, it goes through a process of calculating the criteria for frequency components and amplitudes that need to be responded to the attribute. From the data, the local maximum technique is used to transform the data to FFT spectrum, as shown in Equation (2), and all peak components of the FFT Spectrum are found by comparing the y(i) value with the previous value and the next value, y(i−1) and y(i+1), respectively. After calculating diagnostic variables such as 1X components and GRF components based on the input facility information, the components that match the detected peak components and calculated diagnostic variables are extracted, and the final recognition is performed by determining which reference value is included [24].

(2) y(i)>y(i−1) & y(i)>y(i+1)

#### 3.2.2. Haystack Energy Recognition

**Figure 12:**
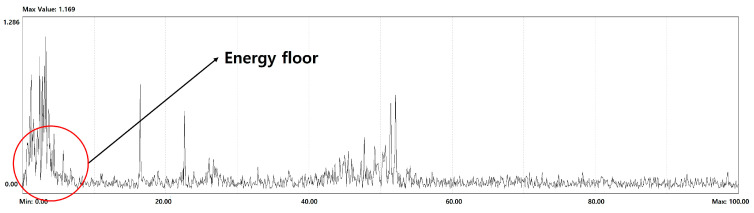
Example of Energy Floor in FFT Spectrum.

**Figure 13:**
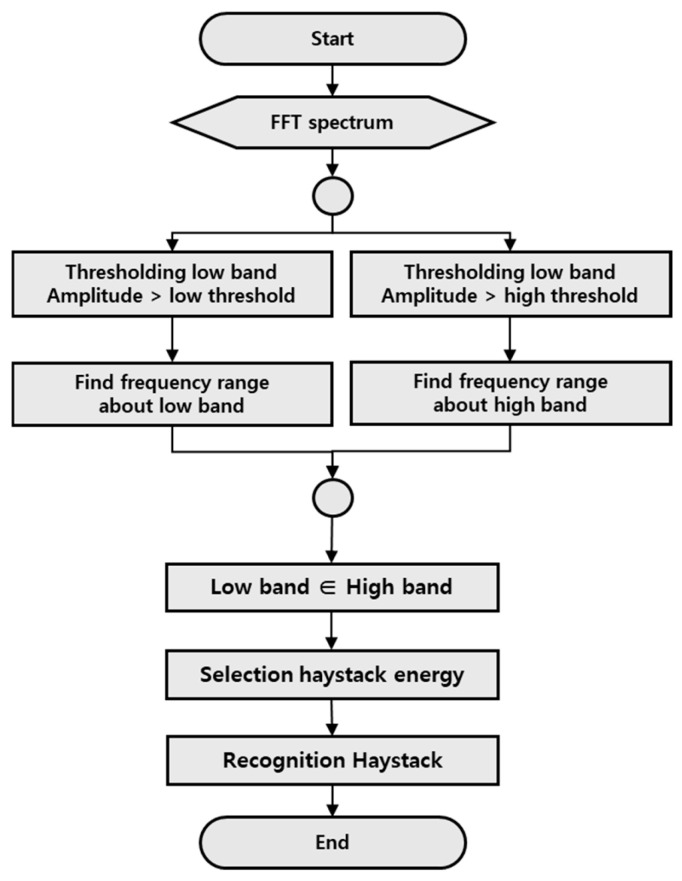
Flowchart of Energy Floor Recognition Algorithm.

Figure 12 is the FFT spectrum showing an example of haystack energy occurring. In the haystack energy, the peak does not appear continuously and appears in the form of a floor, and the frequency range or the size of the component is not constant. To recognize this, it is necessary to distinguish it from the noise component and consider the amount of energy, the frequency interval generated, and finally, how long the frequency interval occurs. In other words, it means that it is necessary to recognize whether a component having a certain reference value or more that is distinguished from a noise component appears continuously. Figure 13 shows the process of haystack energy detection technology in an algorithmic flow chart. An algorithm was developed based on the principle of detecting for the haystack energy using the minimum threshold to distinguish between the haystack energy and the noise component, and the maximum threshold to identify the continuous area in the form of the haystack. First, the minimum range was set for the range exceeding the minimum threshold value distinguished from noise, and the continuous range was obtained as the candidate range for the haystack energy. For the candidate range obtained using the minimum threshold value, a range exceeding the maximum threshold value was searched, and the range satisfying the minimum range of the range was estimated as the haystack energy. In other words, the algorithm was configured to distinguish the haystack energy by setting the criteria for values exceeding the maximum range to be judged as faults on the FFT as each threshold to determine that the value exceeding the minimum range is not noise. Therefore, if the energy value of the detected haystack is obtained from the algorithm and the reference value is exceeded, it may be determined that the haystack energy exists. It can also respond to attributes based on information about the extent to which the haystack energy exists.

#### 3.2.3. Impulse Component Recognition

**Figure 14:**
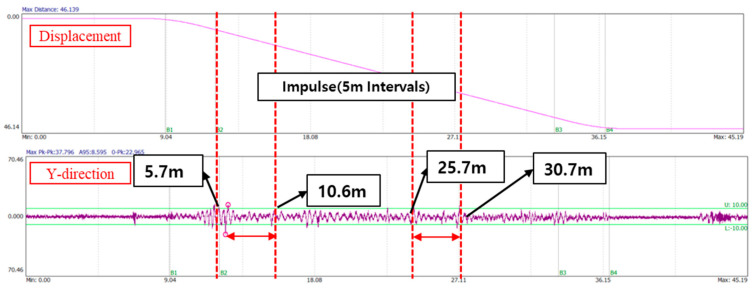
Examples of Impulse Events Caused by Guide Rail Misalignment.

Faults that require recognition of impulse components are faults related to guide rails, and although the length of the installation may vary slightly depending on the country, it is set to be recognized at 5 m intervals based on Korea. Figure 14 is a time waveform signal showing the impulse component generated when the guide rail is misaligned, and although it occurs at intervals of 5 m, it does not occur at all guide rail locations. Based on these characteristics, it is necessary to check whether the connected location of the guide rail and the location of the impulse component coincide. As shown in Figure 14, it occurs at points 5.7 and 10.6 m, and at 25.7 m and 30.7 m. In other words, it is necessary to examine whether the peak of the impulse component exceeds the threshold and whether the corresponding occurrence location coincides.

**Figure 15:**
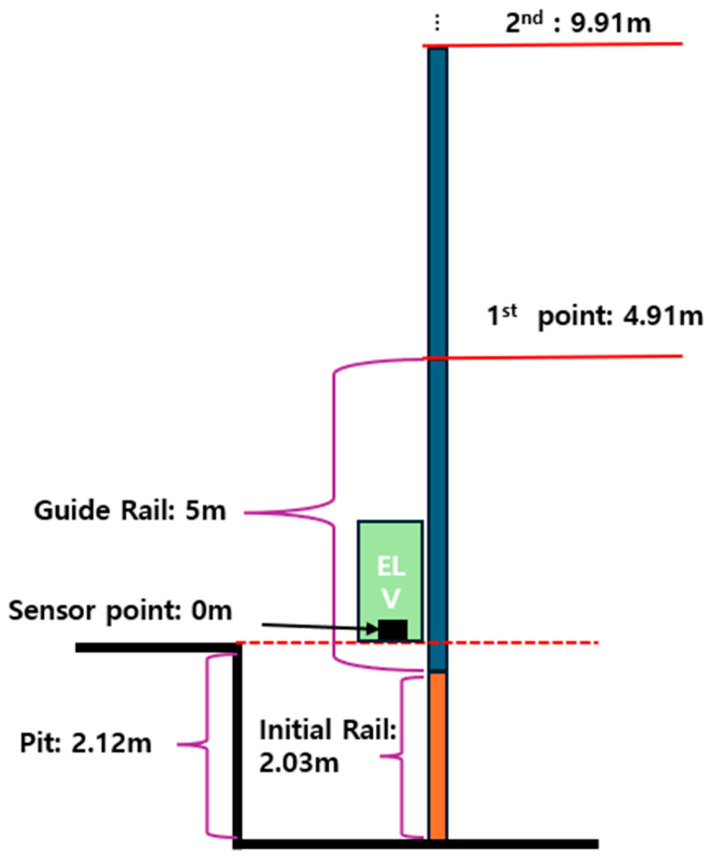
Schematic Diagram of Guide Rail Installation.

Because the joint location of the guide rail is not simply 5 m apart, the actual distance from the measured location should be considered, as shown in Figure 15. Equation (3) is an equation for obtaining the location of the first guide rail joint, calculated by subtracting the difference between the pit depth and the initial rail length from the guide rail length. In addition, the entire joint location can be calculated by reflecting the fact that the guide rails are installed at 5 m intervals, and the bracket location can also be calculated in the same way. Using this information, the section where the Peak-to-Peak value exceeds the threshold was searched, and the error between the measured pit depth and initial length was considered, and it was set to be recognized by applying an error range of ±5% to the calculated value.

(3) $$A_{1st\;joint}=L_{guide}-(D_{pit}-L_{initial})$$

*where* $A_{1st\;joint}:\;Joint\;Location\;of\;First,$

$$L_{guide}:Guide\;rail\;length,$$

$$D_{pit}:Pit\;depth,$$

$$L_{initial}:Initial\;Guide\;rail\;length.$$

#### 3.2.4. Acceleration Change Recognition

**Figure 16:**
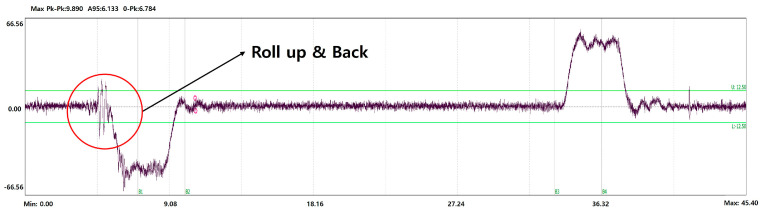
Example of Roll Up & Back.

**Figure 17:**
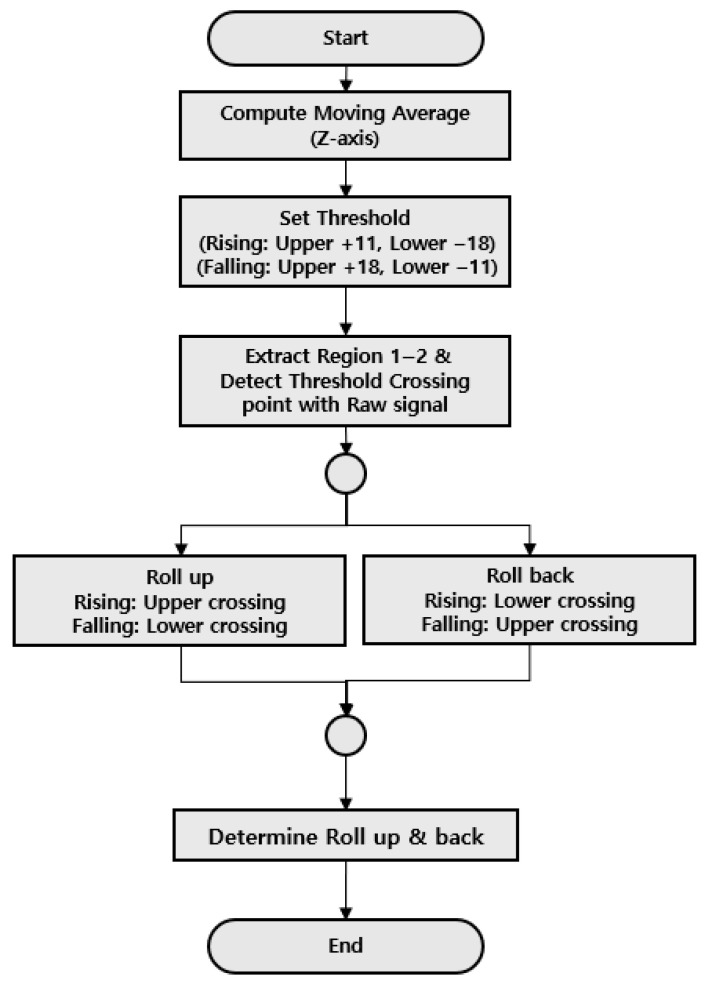
Flowchart of Acceleration Change Recognition Algorithm.

To recognize the impulse component in the acceleration or deceleration section of the operation, it must be distinguished from external noise or components generated by closing the door. Figure 16 is a time waveform showing the impulse component that occurred just before operation. Since it occurs just before acceleration or deceleration, extraction is required between Regions 1 and 2 of Section 3.1 to recognize it and must be distinguished from peak components caused by mechanical faults, not just extracting impulse components. Figure 17 shows the overall algorithm for recognizing abnormal signals before acceleration or deceleration. To generate the threshold, the moving average is calculated based on the Z-axis signal and then the upper threshold and the lower threshold are set based on this. Then, the analysis range is set by extracting the 1–2 section, which is the region before acceleration or deceleration occurs. After comparing the set threshold with the raw signal to detect for crossing, determine and recognize which threshold is exceeded, upper or lower.

## 4. Verification of Recognition Technology

**Figure 18:**
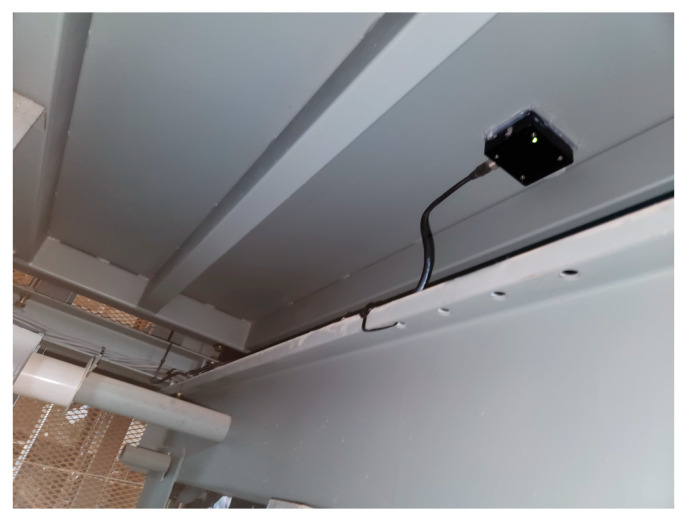
Sensor installation location (elevator bottom section).

To apply recognition technology to rule-based diagnosis, the object to be recognized must be clearly entered according to the criteria so that the attribute about the discriminant can be responded. Therefore, in this study, verification was performed on four recognition targets for the same facility, and the previously set threshold was adjusted up and down because it was to check how clearly recognition was made, not to diagnose faults. Facility and operation information are summarized in Table 3, which is essential for diagnosing the traction machine, guide roller, and guide rail. If there is no information on the guide roller and guide rail, diagnosis may be performed only for the traction machine and operation, and other information is essential input information for diagnosis. The sensor and measurement equipment specifications are summarized in Table 4. Vibration data were acquired using a triaxial acceleration sensor mounted at the lower part of the elevator car, as illustrated in Figure 18. The sensor installation position was selected to ensure stable signal acquisition while capturing vibration components relevant to traction and guidance mechanisms.

Data were collected during a single non-repetitive travel from the lowest floor to the highest floor without passengers. The number of samples for each measurement was fixed at 256 Hz, and no repeated measurements were performed. This measurement strategy reflects practical field constraints and focuses on capturing representative operational vibration characteristics rather than averaging repeated trials. Sensor calibration was performed prior to measurement, and the calibration error was maintained within ±5%. No additional artificial noise filtering was applied beyond standard signal conditioning, ensuring that the acquired data reflect realistic operating conditions and preserving the reliability of fault-related vibration features.

In elevator condition monitoring, sensor mounting locations are clearly specified by international standards and national inspection guidelines. These standards define not only the physical locations for sensor installation but also the measurement directions and operating conditions, ensuring consistency and reproducibility across different facilities. Since the proposed diagnostic framework follows these standard-compliant sensor configurations, the diagnostic results are not significantly affected by variations in sensor placement. Moreover, the system is designed to operate under real service conditions, explicitly incorporating operational noise and background vibrations generated during elevator operation.

**Figure 19:**
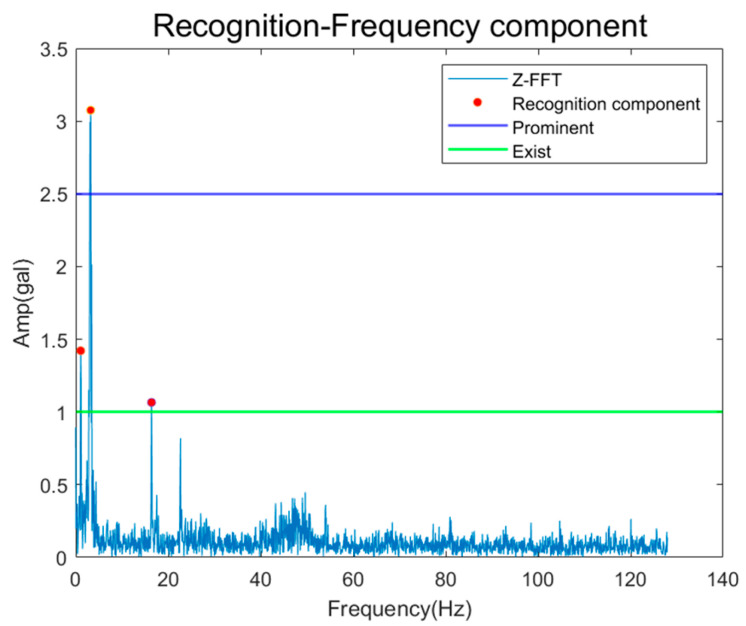
Results of Frequency Component Recognition.

The vibration amplitude evaluation criteria associated with elevator diagnostics are defined based on regulatory and institutional standards, which may differ across countries and jurisdictions. Such differences reflect variations in safety regulations and evaluation policies rather than methodological ambiguity. In this study, amplitude thresholds are applied as diagnostic reference levels rather than absolute safety limits, enabling consistent interpretation of vibration severity within each regulatory framework. Specifically, the proposed method follows the vibration evaluation criteria provided by the Korea Land & Housing Corporation (LH). As a result, the proposed method maintains robustness across different elevator systems by aligning amplitude-based evaluations with standardized, region-specific criteria. Verification was performed on the Z-axis signal in which the traction machine and motor-related fault frequencies were generated. As shown in Figure 19, as a result of checking the components recognized in the FFT spectrum, one Prominent component and two Exist components were recognized, and Operating speed (1X) and triple (3X) components appeared, and additional Supply frequency components appeared. Table 5 shows the error rates for the recognized and calculated results. It is confirmed that an error of about 2–2.7% occurs, which is estimated to be the difference caused by the number of lines in the FFT spectrum and the sampling rate.

**Figure 20:**
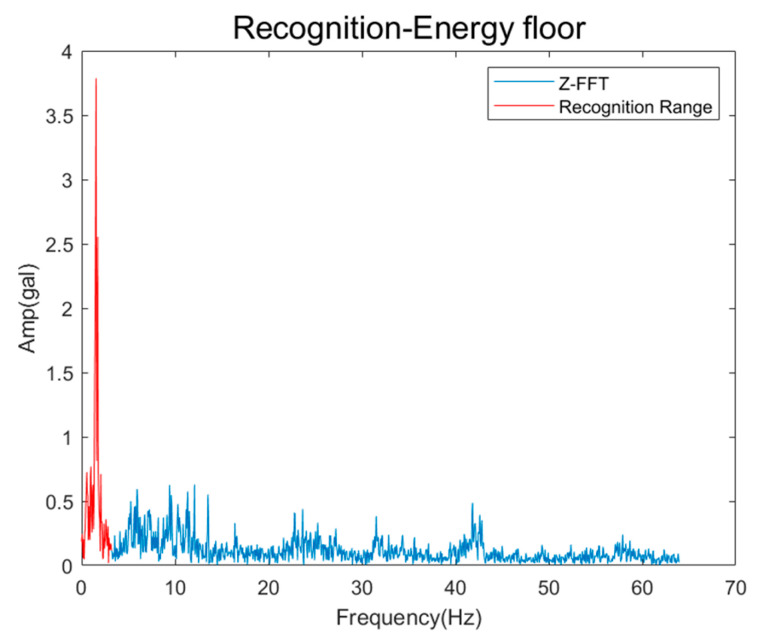
Results of Energy Floor Recognition.

Next, it was determined whether or not to recognize the faults in which the energy floor occurred. Figure 20 shows the FFT spectrum in which the energy floor was recognized, and Table 6 shows the recognized frequency range and energy density of the energy floor. The frequency range is 0–3.21 Hz, but when visually examined, it appears to extend to 12 Hz, but this is caused by noise, and if closely checked, it can be determined that the energy floor does not occur to 12 Hz with a value close to 0 at around 3.8 Hz. Additionally, energy density is not an indicator of the presence or absence of a fault, but it is a value that can be used to determine the degree of severity of the fault.

**Figure 21:**
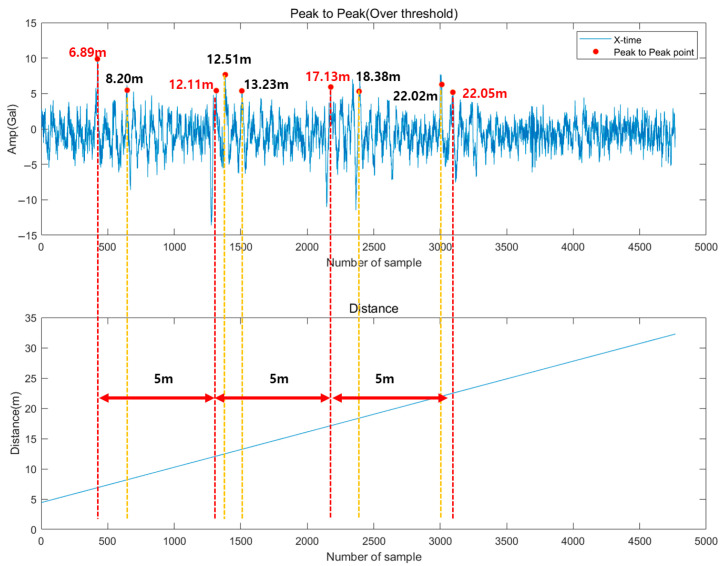
Matching of Joints and Brackets with Impulse Components.

For the recognition of the impulse component, the point where the guide rail is connected and the bracket location should be checked first. Table 7 summarizes the joint and bracket locations, and the joint is installed at intervals of 5 m starting from 2.02 m. The brackets are installed in two locations on one rail, and the spacing attached to each rail is the same, so they were calculated at 5 m intervals. Figure 21 is a graph showing the X-axis time waveform and distance, and it was confirmed that the Peak-to-Peak component and the guide rail joint coincided at four points from Point 2 to Point 5. The recognized results are summarized in Table 8, and since the error of the previously recognized interval was set to ±5%, it is confirmed that all Peak-to-Peak in between were detected. The error rate has a value of less than 2%, and it can be said that it is clearly recognized if there is no error in the measured initial length and pit depth of the guide rail.

**Figure 22:**
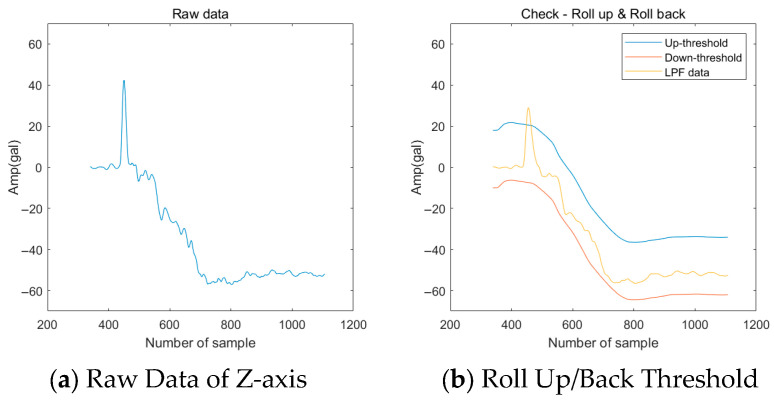
Roll-Up & Roll-Back Recognition Results.

Figure 22a is Z-axis raw data, and Figure 22b is a diagram showing the Up/Down threshold using moving averaging after LPF. The fault was recognized as a Roll back because it was recognized by Up threshold at the start of the falling section, and it is only estimated which threshold was exceeded first. The recognized results can confirm that they are clearly recognized.

## 5. Verification of Rule-Based Diagnosis

**Figure 23:**
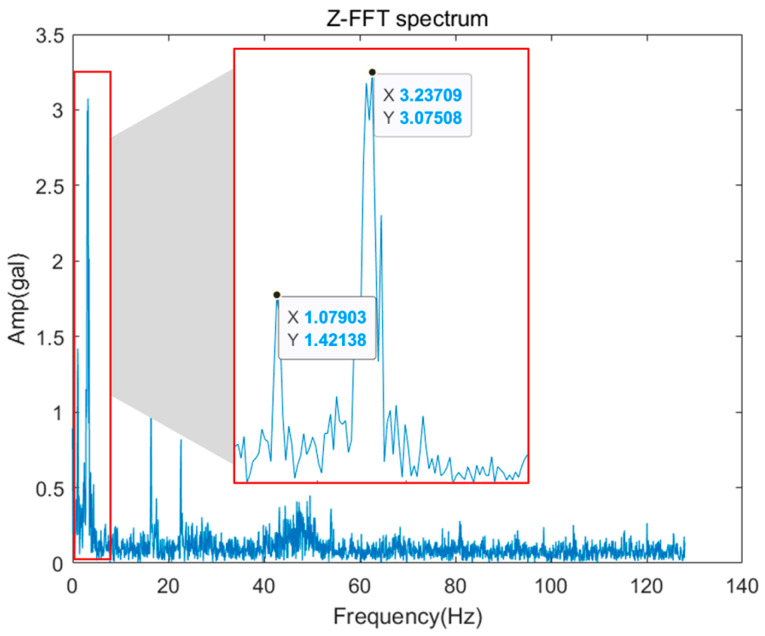
Misalignment Fault Frequency Components.

**Figure 24:**
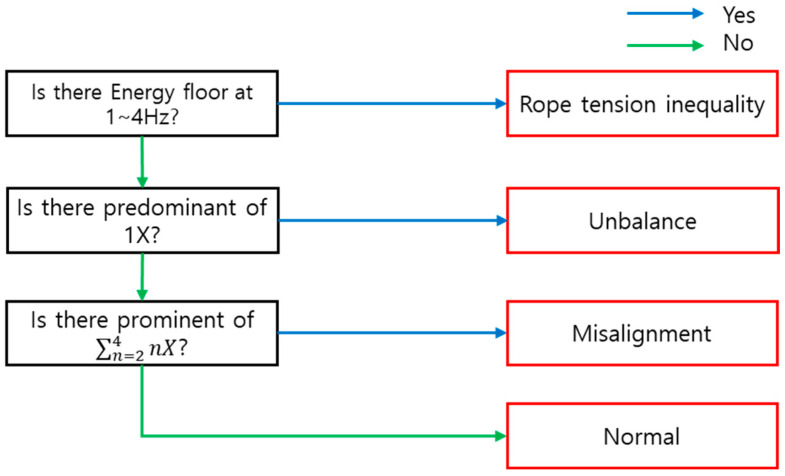
Decision Tree of Main Sheave Diagnosis.

As mentioned earlier, rule-based diagnosis draws conclusions from generated attributes and responses to them. In this study, five decision trees were generated, but only one example was shown for the traction machine. Figure 23 is an FFT showing a fault where misalignment occurs, and the operating speed of 1.12 Hz and the corresponding 3X component responding to it occurs. Table 9 shows the attributes organized in consideration of causality in Table 2, and the response to this is that the 1X component is 1.42 gal, so it does not belong to Predominant. Next, the sum of the 2–4X components exceeds 2.5 gal, which belongs to Prominent and responds to it, and finally, the energy floor was not recognized and the response was not performed. Based on this, if the result is derived according to the decision tree as shown in Figure 24, it is diagnosed as a condition of traction machine misalignment.

**Figure 25:**
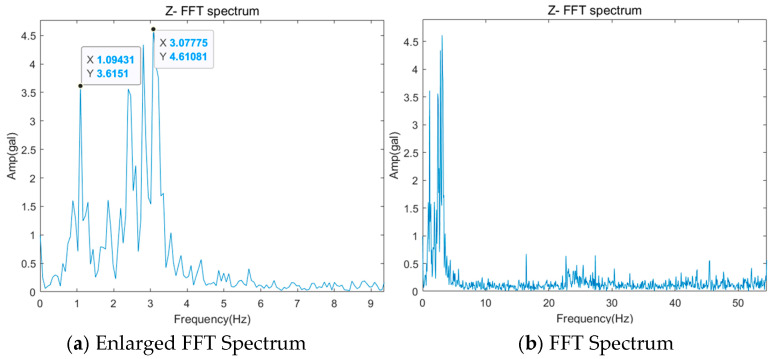
FFT Spectrum of Data Set 4.

In this way, verification was performed with 10 data from the same facility. As a verification method, the acquired data were analyzed and diagnosed, labeled, and the results were compared by operating the algorithm. Table 10 shows the results of analysis for one facility, for which diagnosis was not performed because there was no information on the motor. As a result of diagnosis, it was confirmed that all fault conditions that met the recognition target were shown, but as a result of comparison, it was confirmed that there was a slight difference from the diagnosis result in the traction machine of Data No 4. To perform additional analysis, the FFT spectrum corresponding to Figure 25 is shown. When the frequency components were analyzed by magnification, the energy floor appeared in a non-continuous form and was diagnosed as misalignment. On the other hand, when the recognition algorithm was used, it is estimated that the haystack energy is recognized because the verification is performed in the entire frequency range, and the section higher than the noise continues. Overall, it was confirmed that the diagnosis was made based on the factors used by experts in the analysis, so almost identical results were derived, and additional problems such as human errors are not expected to occur because diagnosis is performed according to the criteria for fault conditions that cause confusion.

In addition, this study deliberately adopts a rule-based diagnostic framework. When fault signatures are simple and clearly distinguishable when each fault type is characterized by independent and well-defined frequency components—traditional rule-based or model-based approaches are sufficient and often preferable. In contrast, complex compound faults involve multiple fault frequencies interacting simultaneously, leading to subtle and high-dimensional patterns that are difficult to generalize reliably across different elevator types.

Although advanced methods such as SVMs or deep learning models can be effective in certain scenarios, their application requires large and comprehensive labeled datasets that cover a wide variety of fault combinations and operating conditions. In practical elevator systems, however, the number of possible fault types and their combinations is extremely large, making exhaustive data collection and robust multi-class classification infeasible. For this reason, a purely data-driven classification of compound fault states may not be suitable at the current stage. Therefore, the proposed rule-based approach focuses on transparent, physics-consistent interpretation of dominant frequency components, aligning closely with expert diagnostic logic. This strategy ensures robustness, interpretability, and practical applicability in real elevator maintenance environments, while providing a scalable foundation that can be extended to more complex hybrid or data-driven approaches in future work.

## 6. Conclusions

In this study, rule-based diagnostic automation technology was developed for elevators to improve the problems of experience and visual inspection, which are existing elevator maintenance methods, and breakdown maintenance methods. The time waveform and FFT spectrum of the vibration signal were used to effectively detect and analyze possible faults for the main components of the elevator. The main component targets were divided into five main regions: driving part, motor, guide rail, car, and guide roller, and the fault patterns characteristic of the vibration signal for each component were analyzed and organized.

In addition, four recognition technologies were developed and applied to compare and analyze them so that rule-based diagnosis can be automatically responded to. First, we detected for all peaks appearing in the input information and FFT spectrum and matched the corresponding components to perform frequency component recognition, thereby enabling us to detect the fault of frequency components. Secondly, to detect haystack energy caused by random excitation rather than peak components, two thresholds were applied to detect and distinguish noise, differences, and how continuously energy is generated [24]. Third, considering the characteristics of impulses occurring at regular intervals on the guide rail, an impulse component detection technique capable of recognizing the condition at the joint and bracket locations of the guide rail was developed. Finally, to detect abnormal signals that occur before the start of the acceleration or deceleration section of the elevator, the lower/upper threshold was applied using the moving average to enable the detection of faults for Roll up & Roll back.

These recognition technologies were developed to build a rule-based automatic diagnostic system, which was applied to 10 different units in the same facility to compare and analyze the results diagnosed by experts. The rule-based diagnosis expert system was confirmed to produce more than 99% identical results because it performs diagnosis according to rules set by experts. It was confirmed that different results occurred from one fault, but this is thought to be because the driving part side occurred at a relatively low frequency, so the expert analyzed it in an expanded manner. When checking the entire spectrum, it is confirmed that a continuous energy floor is observed in a section higher than the noise, and it is estimated that human error can be reduced.

Through this, we developed a technology that complements the data shortage problem of existing AI-based diagnosis methods while enabling customized diagnosis suited to the elevator operating environment. The rule-based diagnostic system developed in this study was able to detect faults with higher reliability than existing maintenance methods, and automatically analyze elevator faults without relying on the experience of maintenance personnel. In addition, by presenting a diagnostic technique that reflects the structural characteristics of the elevator, a methodology differentiated from the conventional diagnostic technique for general rotating bodies was applied, and a rule-based approach to solve the data shortage problem was used to compensate for the shortcomings of AI-based methods and increase compatibility with existing maintenance systems. The rule-based elevator diagnosis technology presented in this study is evaluated as a practical methodology that can contribute to maximizing the efficiency of elevator maintenance and building a safer elevator operating environment. It will be important to continuously improve technology through future research and develop research in the direction of increasing applicability in actual industrial sites.

## Figures and Tables

**Table 1:** Motor Specification.

Motor Information	
Poles	20
Supply frequency	24.8 Hz
Rotating frequency	2.48 Hz
REV	148.8 r/min

**Table 2:** Fault Condition and Characteristics by Region.

Diagnosis Region	Fault Condition	Direction	Domain	Features
Driven	Unbalance	Z	FFT spectrum	1$X=\frac{RPM}{60}$ or $\frac{V_{car}\;R}{\pi D_{sheave}}$ (Drive speed)
	Misalignment	Z	FFT spectrum	2X or 3X (Rotating speed)
	Rope Tension Inequality	Z	FFT spectrum	Energy floor (1~4 Hz)
Motor	Eccentricity Faults	Z	FFT spectrum	$f_{eccen}=f_{s}\left(1\pm \frac{k}{p}\right)$
	Magnetic Faults	Z	FFT spectrum	$f_{dmg}=f_{s}\left|1\pm \frac{k}{p}\right|$
	Slot Faults	Z	FFT spectrum	$f_{slot}=f_{s}\left(1\pm \nu \frac{Z_{slot}}{p}\right)$
	External Electrical Fault	Z	FFT spectrum	$f_{s}$
Guide Rail	Guide Rail Misalignment	X or Y	Time waveform	Shock occurs at 5 m intervals (Guide rail intervals)
	Guide Rail Support Faults	X or Y	Time waveform	Shock occurs at 2.5 m intervals (Guide rail support intervals)
Drive	Roll Up	Z	Time waveform	Impulse vibration occurs in the same direction as the acceleration section before the acceleration section in the time waveform
	Roll Back	Z	Time waveform	Impulse vibration occurs in the opposite direction as the acceleration section before the acceleration section in the time waveform
Guide Roller	Guide Roller Interference	X or Y	FFT spectrum	Guide roller frequency 1X
	Guide Roller Misalignment	X or Y	FFT spectrum	Guide roller frequency 2X or 3X

**Table 3:** Elevator Machinery Data.

Elevator Information	
Operating speed (RPM, Hz)	67 (1.11)
Car velocity (m/s)	1.75
Roping ratio	2:1
Sheave diameter (mm)	500
Guide roller diameter (mm)	125
Guide rail initial length (mm)	2030
Pit depth (mm)	2120
Supply frequency (Hz)	16.7
Number of pole (EA)	30

**Table 4:** Sensor and Measuring Equipment Specifications.

	Information	Value
Sensor	Type	Piezo-resistive MEMS
	Direction	X, Y, Z (3-axis)
	Range	6 g, 8 g, 10 g, 20 g
ADC (Measuring Equipment)	Bandwidth	70 kHz
	DC accuracy	9.8 µV/°C, 1.8 ppm/°C
	SNR	111 dB, 52 kSPS
Measurement	Sampling rate	256 Hz
	Duration	35~50 s

**Table 5:** Error Analysis of Frequency Component Recognition.

	Recognized FFT Spectrum Component			Calculated Hz Value	Error Rate
Rank	Criteria	Amplitude (gal)	Frequency (Hz)	Frequency (Hz)	
1	Prominent	3.08	3.24	3.33 (3X)	2.7%
2	Exist	1.42	1.08	1.11 (1X)	2.7%
3	Exist	1.07	16.38	16.7 (Supply freq.)	2%

**Table 6:** Results of Energy Floor Region.

	Frequency (Hz)	Energy Density
Energy Floor Region	0–3.21	93.20

**Table 7:** Computed Location of Joints and Brackets.

	Location Number							
	1	2	3	4	5	6	7	8
Joint	2.02	7.02	12.02	17.02	22.02	27.02	32.02	37.02
Bracket(1)	4.19	9.19	14.19	19.19	24.19	29.19	34.19	39.19
Bracket(2)	5.86	10.86	15.86	20.86	25.86	30.86	35.86	40.86

**Table 8:** Error Analysis of Impulse Component Recognition.

Location Number	Expected Location	Recognition Location	Error Ratio
2	7.02	6.89	1.9%
3	12.02	12.11	0.7%
4	17.02	17.13	0.6%
5	22.02	22.05	0.1%

**Table 9:** Rule-based Diagnosis Attribute and Response.

	Attribute	Response
1	Is there predominant of 1X?	N
2	Is there prominent of $\sum_{n=2}^{4}nX$?	Y
3	Is there Energy floor at 1~4 Hz?	N

**Table 10:** Results of Rule-based Diagnosis Implementation.

Data No.	Recognition Result				Analysis Result			
	Traction Machine	Guide Roller	Guide Rail	Driven	Traction Machine	Guide Roller	Guide Rail	Driven
1	Normal	Load	Misalign.	Roll up&back	Normal	Load	Misalign.	Roll up&back
2	Normal	Normal	Normal	Roll up&back	Normal	Normal	Normal	Roll up&back
3	Normal	Normal	Normal	Roll up&back	Normal	Normal	Normal	Roll up&back
4	Rope tension	Normal	Normal	Normal	Misalign.	Normal	Normal	Normal
5	Normal	Normal	Normal	Roll up&back	Normal	Normal	Normal	Roll up&back
6	Normal	Load	Misalign.	Roll up&back	Normal	Load	Misalign.	Roll up&back
7	Misalign.	Misalign.	Normal	Normal	Misalign.	Misalign.	Normal	Normal
8	Normal	Load	Misalign.	Roll up&back	Normal	Load	Misalign.	Roll up&back
9	Misalign.	Load	Misalign.	Normal	Misalign.	Load	Misalign.	Normal
10	Normal	Misalign.	Normal	Normal	Normal	Misalign.	Normal	Normal

## Data Availability

The data presented in this study are available on request from the corresponding author.
